# Anti-Psoriatic Effects of Antimony Compounds In Vitro

**DOI:** 10.3390/molecules26195814

**Published:** 2021-09-25

**Authors:** Fabian Gendrisch, Birgit Haarhaus, Christoph M. Schempp, Ute Wölfle

**Affiliations:** Research Center Skinitial, Department of Dermatology, Medical Center-University of Freiburg, Faculty of Medicine, University of Freiburg, 79104 Freiburg, Germany; birgit.haarhaus@uniklinik-freiburg.de (B.H.); christoph.schempp@uniklinik-freiburg.de (C.M.S.); ute.woelfle@uniklinik-freiburg.de (U.W.)

**Keywords:** psoriasis, keratinocytes, dendritic cells, antimony, inflammation

## Abstract

Psoriasis is a chronic inflammatory skin disease characterized by hyperproliferation of keratinocytes and a pro-inflammatory milieu in the skin. While patients with moderate to severe psoriasis are treated using targeted therapies (small molecules and monoclonal antibodies), patients suffering from milder forms are still in need of effective topical products without adverse effects. Antimony compounds (ACs) are regularly used as anti-inflammatory compounds in traditional and anthroposophic medicine and as antiprotozoan drugs. Here, we examined the effect of metallic antimony, natural antimony(III) sulfide and potassium antimonyl(III) tartrate in vitro on psoriasis-like keratinocytes and the human dendritic cell line THP-1 using qPCR, immunocytochemistry, ELISA and flow cytometry. In psoriatic keratinocytes, ACs inhibited the overexpression of the antimicrobial peptide β-defensin 2 and glucose transporter 1, as well as the hyperproliferation marker keratin 17. Furthermore, ACs mediated anti-inflammatory effects by reducing nuclear translocation of the p65 subunit of NF-κB and pSTAT3 and inhibited pro-inflammatory cytokine secretion by keratinocytes. In addition, ACs displayed anti-psoriatic effects by reducing the activation of IFN-α-treated THP-1 cells as well as the expression of the psoriasis-promoting master cytokine IL-23 by these cells. While all ACs showed anti-psoriatic effects, the most prominent results were seen with potassium antimonyl(III) tartrate. In summary, ACs display numerous anti-psoriatic effects in vitro at subtoxic concentrations. We conclude that ACs are interesting compounds for the topical treatment of psoriasis that warrant further investigation in clinical studies.

## 1. Introduction

Psoriasis is a chronic inflammatory skin disease with an estimated global prevalence of 2–3% [1]. Plaque-type psoriasis (or psoriasis vulgaris) is the most common manifestation accounting for about 90% of all cases [2]. Typical signs are thickened erythematous and scaly plaques of varying size. Histological features include infiltration of the skin with mononuclear inflammatory cells and neutrophil granulocytes, and epidermal hyperplasia with disturbed terminal differentiation of keratinocytes, as expressed by retention of the nucleus in the normally anucleated cells in the stratum corneum (parakeratosis). Psoriasis may cause itching and pain, resulting in psychiatric comorbidities such as depression.

Psoriasis is triggered by an interplay of endogenous and exogenous factors together with a genetic predisposition. Triggers include injury of the skin by, for example, scratching or mechanical stress at the site of the lesion (Koebner phenomenon). In addition, infections with certain bacteria (e.g., streptococci) or various drugs (e.g., β-blockers, lithium) may trigger psoriasis. The pro-inflammatory cascade is initiated by the release of self-nucleotides from damaged keratinocytes. These self-nucleotides can form complexes with antimicrobial peptides (AMPs) such as the cathelicidin-derived LL-37 or β-defensin 2 (BD2), leading to the activation of innate immune cells such as dendritic cells (DCs) via the binding of these complexes to Toll-like receptors [3]. This initiates a pro-inflammatory signaling cascade resulting in activation of naïve CD4^+^ T cells followed by their proliferation and differentiation into different T helper subtypes (T_H_ cells). Cytokines such as tumor necrosis factor α (TNF-α), interleukin (IL)-12 and IL-23 play a key role in this inflammatory process [4,5]. In response to these factors, different T cell subtypes secrete pro-inflammatory cytokines leading to progression and perpetuation of the disease. These cytokines include IL-22 from T_H_22 cells as well as IL-17A secreted by T_H_17 cells [6,7]. While IL-22 is responsible for the characteristic epidermal hyperplasia and reduced differentiation of keratinocytes accompanied by parakeratosis, IL-17A activates important signaling pathways including the Janus kinase/signal transducer and activator of transcription-(JAK/STAT) as well as the nuclear factor kappa-light-chain-enhancer of activated B cells (NF-κB) pathway in keratinocytes. This results in the secretion of several pro-inflammatory mediators (e.g., IL-6 or IL-8) and the expression of typical psoriasis markers such as keratin 17 (*KRT17*) or glucose transporter 1 (*GLUT1*) by the keratinocytes. The uncovering of these pathways led to the development of targeted therapies, i.e., monoclonal antibodies against TNF-α, IL-12/23, IL-23 and IL-17A [8,9,10]. However, these biologicals are only used in cases of severe psoriasis while most patients (~90%) suffer from mild to moderate psoriasis. This group is usually treated with topical therapies such as corticosteroid or dithranol, in some cases combined with phototherapy. While the topical substances are effective, their use is limited either because they are not suitable for long-term use due to adverse effects or because safe handling is difficult and often requires inpatient treatment in specialized hospitals. Therefore, outpatients could benefit from new topical treatment options that may replace topical drugs with possible adverse effects. Natural substances used in traditional medicine for the treatment of inflammatory diseases are interesting candidates to potentially take this role.

Interesting substances in this category are the metalloid antimony and its chemical derivatives. Antimony has a long-standing tradition of therapeutical usage reaching back to the 16th century [11]. Even today, drugs containing antimony compounds (ACs) are regularly used to treat various diseases. However, in addition to its beneficial effects, antimony uptake can also have detrimental consequences. It plays a role in environmental contamination of drinking water and soil used to grow nutritional plants. Antimony is brought into circulation by the distribution of industrial sewage water, motor vehicle emissions or as a part of pesticides. The concentration of antimony in food and environment is strictly limited, as the oral uptake of higher concentrations, especially in cases of chronic exposure, might lead to detrimental effects to the health of humans. In earlier times, ACs were thought to be carcinogenic. However, carcinogenicity of ACs could not be confirmed with oral uptake, and only very limited evidence exists for carcinogenicity of ACs from inhalation studies in animals [12]. The limit of the tolerable daily oral uptake of antimony was set to 6 µg/kg body weight by the World Health Organization. The ‘no observed adverse effect level’ (NOAEL) is 6 mg/kg body weight. However, as psoriasis in its milder forms mostly affects the skin, topical application could give good results without the risk of adverse effects due to oral uptake.

The most known application of ACs is the treatment of parasitic diseases such as leishmaniasis [13] or schistosomiasis [14]. However, ACs are also frequently utilized in anthroposophic and ayurvedic medicine [15,16], for example, to treat diseases such as inflammatory bowel disease and inflammatory skin diseases such as rosacea, eczema and psoriasis [17,18,19]. However, no clinical or experimental studies have confirmed the efficacy and possible mode of action of ACs in these conditions. The beneficial effects of ACs in these diseases might be mediated by a modulation of pro-inflammatory cell signaling as shown for antimony chloride in T cells at sub-toxic concentrations [20].

Nevertheless, psoriasis is not only characterized by a prominent T cell response but also by enhanced proliferation and reduced differentiation of keratinocytes together with pro-inflammatory signaling. Therefore, psoriasis patients might not only benefit from potential immunomodulatory effects but also from anti-proliferative properties of ACs. The latter have been demonstrated in cancer cells [21] but might also be useful in psoriasis. The topical application of ACs can avoid potential adverse effects caused by systemic exposure.

Therefore, the aim of our work was to investigate potential anti-psoriatic effects of three different ACs that are regularly used in anthroposophic and ayurvedic medicine. The compounds are antimony (Sb, also known as stibium), natural antimony(III) sulfide (Sb_2_S_3_, also known as stibnite or antimonite) and potassium antimonyl(III) tartrate (K_2_Sb_2_C_8_H_4_O_12_·3 H_2_O, also known as tartarus stibiatus or tartar emetic). While antimony (A) and natural antimony(III) sulfide (AS) are already used in anthroposophic medicine to treat dermatological disorders, potassium antimonyl(III) tartrate (PAT) is used to support the healing of bronchitis, bronchial asthma or pulmonary fibrosis [22]. A major part of our experiments was performed with in vitro generated psoriasis-like keratinocytes because they initiate and maintain pro-inflammatory signaling in psoriasis and represent the major cell type that causes the clinical phenotype.

In our in vitro model of psoriasis using primary human keratinocytes (PHKs), antimony, natural antimony(III) sulfide and potassium antimonyl(III) tartrate displayed promising anti-psoriatic effects. Not only did they reduce psoriasis-associated gene expression, but also inhibited pro-inflammatory pathways such as NF-κB and STAT3 signaling, eventually resulting in reduced cytokine secretion. ACs also displayed inhibitory effects in a human dendritic cell line stimulated with IFN-α.

## 2. Results

### 2.1. Analysis of AC Cytotoxicity

**Figure 1:**
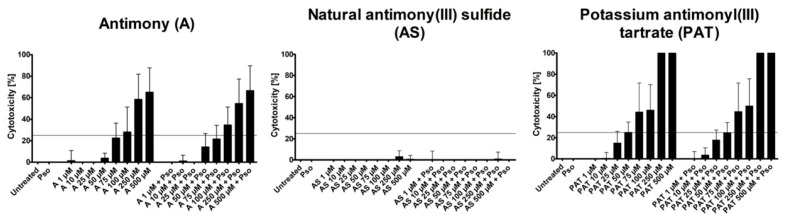
LDH assay to determine cytotoxicity of ACs. PHKs were treated with increasing concentrations of A, AS and PAT for 24 h alone or in addition to stimulation with Pso (20 ng/mL of IL-17A, IL-22 and TNF-α). Afterwards, cell culture supernatants were collected and LDH release was analyzed. Results are shown as mean ± SD of three independent experiments.

As a model for the analysis of the anti-psoriatic effects of ACs, we used psoriasis-like PHKs generated in vitro. These cells show a phenotype similar to keratinocytes from psoriatic lesions [23]. To achieve this phenotype, PHKs were treated with a combination of IL-17A, IL-22 and TNF-α (Pso). First, we analyzed the cytotoxic potential of the substances by measuring the lactate dehydrogenase (LDH) release to identify the ideal concentrations for the following experiments measuring LDH release. The tested concentrations ranged from 1 µM to 500 µM. As both A and AS are not soluble in any solvent suitable for cell culture, concentrations were limited to this value to exclude a high level of particulate matter in the culture medium that could exert a negative effect on the PHKs. Both A and PAT showed an increase of toxicity with growing concentrations, while AS caused no increase of toxicity in all tested doses (Figure 1).

This was the case for cells with or without additional Pso stimulation. However, as we aimed to identify anti-psoriatic effects of the compounds without severe toxic effects that could prevent their usage as a topical treatment, we limited the cytotoxicity of the subsequently used concentrations to 25%, leaving 75% viable cells after Pso stimulation. The highest concentrations still below this mark were 75 µM for A, 500 µM for AS and 50 µM for PAT. We chose those concentrations and added the two next lower concentrations for further experiments, leaving us with 25 µM, 50 µM and 75 µM for A, 100 µM, 250 µM and 500 µM for AS and 10 µM, 25 µM and 50 µM for PAT. As a control, lactose was used because A and AS were supplied as 1:10 trituration in lactose. These triturations are the raw material for subsequent processing in the manufacturing process of antimony-containing products for traditional medicine. Lactose was used in the concentrations corresponding to the ones present in A and AS treatment. In none of the tested concentrations did lactose cause any cytotoxicity (data not shown).

### 2.2. Effect of ACs on Psoriasis Marker Expression

**Figure 2:**
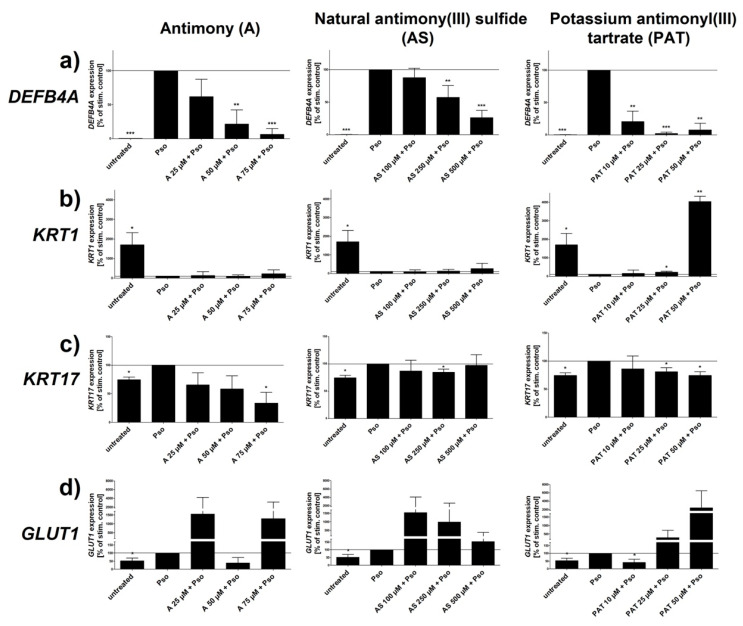
Effect of ACs on Pso-induced gene expression. PHKs were stimulated with Pso (20 ng/mL of IL-17A, IL-22 and TNF-α) for 24 h with or without a pre-treatment of increasing concentrations of A, AS or PAT. (**a**) *DEFB4A*; (**b**) *KRT1*; (**c**) *KRT17* and (**d**) *GLUT1* mRNA expression was analyzed by real-time qRT-PCR with *ACTB* as reference gene for the analysis using the comparative C_T_ method [27]. Values were then normalized setting the Pso-treated PHKs as 100% expression. Results are shown as mean ± SD of three independent experiments. Statistical analysis was performed using column statistics with one-sample *t*-test comparing the samples to Pso stimulation. * *p* ≤ 0.05, ** *p* ≤ 0.01, *** *p* ≤ 0.001.

Several psoriasis markers have been identified and described as characteristic for psoriatic keratinocytes. The AMP BD2 is not only upregulated in psoriatic skin but the expression of its coding gene *DEFB4A* also correlates with the severity of the disease [24,25]. Furthermore, serum levels of BD2 have been shown to be a well suited biomarker for psoriasis [26]. As shown in Figure 2a, all three ACs downregulated the Pso-induced upregulation of *DEFB4A* expression in a dose-dependent manner. Both A and PAT caused the biggest downregulation of gene expression resulting in a level close to that of unstimulated cells. An effect can already be seen at rather low concentrations of 10–25 µM. In contrast, reduction upon treatment with AS starts only at 250 µM, ten times more than A and PAT but still at a lower level of cytotoxicity.

Keratin 1 (*KRT1*), a member of the keratin protein family, is expressed in the epidermis and is involved in the control of the cell cycle as well as differentiation of keratinocytes. As differentiation of these cells is downregulated in psoriatic skin, expression of the early differentiation marker *KRT1* is also reduced [28]. The same holds true for Pso-treated keratinocytes that show a prominent reduction of *KRT1* expression (Figure 2b). However, neither A nor AS were able to revert this effect at any of the tested concentrations. In contrast, PAT was able to upregulate KRT1 expression at a concentration of 25 µM. Using 50 µM resulted in an expression even above the level of untreated cells, reverting the negative effect of Pso stimulation on the differentiation marker. As already mentioned, hyperproliferation is one of the key characteristics of psoriatic keratinocytes. KRT17 is a protein that has been shown to be a marker for hyperproliferation in these cells and was found to be overexpressed in psoriatic skin [29]. As already shown by us, Pso treatment upregulated the expression of *KRT17*, indicating the hyperproliferative phenotype of these cells (Figure 2c). However, PAT, AS and especially A were able to counteract this effect within the set range of toxicity. While PAT and AS were able to reduce the expression to the level of untreated cells, A caused a reduction even below that level. Keratinocyte proliferation—for example, during inflammation and injury—is critically dependent on glucose. GLUT1 is responsible for efficient transport of glucose across the cell membrane into the cytoplasm. Consequentially, *GLUT1* expression was found to be upregulated in psoriatic skin as these cells have an increased need for energy due to the enormous proliferation seen in psoriasis plaques [30]. In addition, it has been described that *GLUT1* expression correlates with disease severity [31]. In line with these studies, Pso treatment led to an increased expression of *GLUT1* in PHKs. However, neither A nor AS were able to reduce *GLUT1* expression significantly. While 50 µM of A showed a tendency towards a reduction, all other concentrations of A and AS even led to a further increased expression. In contrast, 10 µM of PAT led to a decrease of gene expression even below the value of untreated cells while higher concentrations showed the same amplifying tendency as A and AS. The expression of all genes was not influenced by lactose (data not shown).

Taken together, Pso stimulation increased the basal proliferation level and inhibited the early stage of differentiation shown by the reduced expression of the early differentiation marker *KRT1* in untreated cells. Antimony and its compounds AS and PAT influenced psoriasis gene expression in Pso-treated PHKs. Among those, *DEFB4A* and *KRT17* showed the most beneficial changes upon treatment with either A or PAT.

### 2.3. ACs Decrease Inflammation in Psoriasis-like Keratinocytes

**Figure 3:**
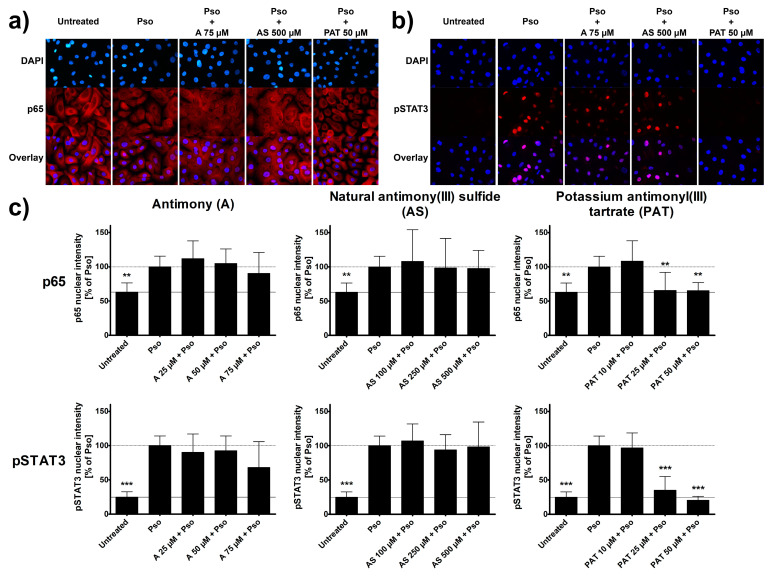
Effect of ACs on the activation of NF-κB and STAT3 signaling pathways. PHKs were stimulated with the Pso cytokine combination (20 ng/mL of IL-17A, IL-22 and TNF-α) for 30 min with or without pre-treatment of A, AS or PAT for 2 h and immunofluorescence staining of the p65 subunit of NF-κB and pSTAT3 were performed. The nucleus is stained with DAPI. Representative pictures of (**a**) p65 and (**b**) pSTAT3 staining in untreated, Pso-treated and AC treated cells. (**c**) The intensity of the staining in the nucleus was measured using the Intensity Ratio Nuclei Cytoplasm Tool (RRID:SCR_018573) in ImageJ. Data shown as mean ± SD of four independent experiments with two pictures per experiment. Statistical analysis was performed using one-way ANOVA with Newman–Keuls post-test comparing the samples to Pso stimulation. ** *p* ≤ 0.01, *** *p* ≤ 0.001.

As psoriasis is an inflammatory disease of the skin, several pro-inflammatory signaling pathways are typically activated in keratinocytes of psoriatic lesions. Among the most important and well-known ones are the STAT3 and the NF-κB pathways [32,33]. Using immunocytochemistry, activation of both pathways was analyzed in psoriasis-like PHKs. Cytokine treatment led to an increased nuclear translocation of the p65 subunit of NF-κB. This is visualized by the disappearance of the shadow in the place of the nucleus that can be seen in untreated cells. Due to the nuclear translocation, the staining shows a similar intensity in the nucleus after Pso stimulation (Figure 3a). In addition, the psoriasis cytokines caused an increased phosphorylation and nuclear translocation of STAT3. While untreated cells show no staining for phosphorylated STAT3 (pSTAT3), stimulated cells show a strong staining in the nucleus (Figure 3b). These findings are confirmed when analyzing the intensity of the staining in the nucleus (Figure 3c). A 2 h pre-treatment with either A or AS had no significant effect on these processes. Even the highest concentrations of 75 µM and 500 µM, respectively, had no visible or measurable difference to Pso-treated cells without pre-treatment. However, PAT was able to reduce the translocation of p65 into the nucleus starting at concentrations of 25 µM which can be seen in the reduction of nuclear staining. Furthermore, PAT not only decreased pSTAT3 nuclear translocation but also the phosphorylation itself, as seen by the lack of staining throughout the cells and not only the nucleus. Lactose had no effect either on p65 translocation or on STAT3 phosphorylation and translocation (data not shown).

**Figure 4:**
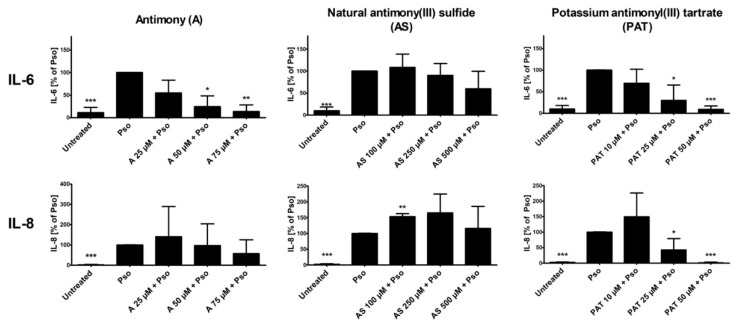
Effect of ACs on Pso-induced secretion of IL-6 and IL-8. PHKs were treated with Pso (20 ng/mL of IL-17A, IL-22 and TNF-α) for 24 h with or without 2 h pre-treatment with A, AS or PAT. Afterwards, cell culture supernatant was collected and secretion of IL-6 and IL-8 were measured using ELISA. Results of four independent experiments are shown as mean ± SD. Statistics analysis was performed using column statistics with one-sample *t*-test comparing the samples to Pso stimulation. * *p* ≤ 0.05, ** *p* ≤ 0.01, *** *p* ≤ 0.001.

In psoriasis, activation of NF-κB and STAT3 in keratinocytes results in the production of pro-inflammatory cytokines such as IL-6 and IL-8 [34,35]. Both cytokines not only increase activation of the keratinocytes themselves, leading to a boost in proliferation, but also contribute to the maintenance of the ongoing inflammation in the skin. We therefore analyzed the effects of A, AS and PAT on the cytokine-induced secretion of IL-6 and IL-8 from PHKs. Again, cytokine secretion of Pso-treated cells was set as 100% and the results of the other sample were calculated in comparison. Pso stimulation led to a great increase of the secretion of both IL-6 and IL-8 by PHKs (Figure 4). While pre-treatment with AS resulted in no significant decrease of IL-6 secretion, a tendency towards a reduced secretion especially at the highest pre-treatment doses is recognizable. However, A and PAT were clearly able to reduce IL-6 levels in the cell culture supernatant dose-dependently with the highest effect at 75 µM or 50 µM, respectively, reducing IL-6 secretion nearly to the level of untreated cells. Looking at IL-8 levels, A shows no significant effect. There was only a reducing tendency towards the highest dose of 75 µM. Surprisingly, pre-treatment with AS did not reduce IL-8 secretion but led to an increase of cytokine secretion. Finally, PAT again had the most prominent anti-inflammatory effect by reducing IL-8 secretion to the level of untreated cells at 50 µM. Again, lactose treatment showed no effect (data not shown).

In summary, while A and AS are limited in their anti-inflammatory capabilities concerning NF-κB, STAT3 and cytokine secretion, PAT also showed promising results in the inhibition of pro-inflammatory signaling in psoriasis-like PHKs, as seen by a reduction of NF-κB and STAT3 pathway activation and reduction in pro-inflammatory cytokine release.

### 2.4. ACs Reduce DC Activation and IL-23 Expression

**Figure 5:**
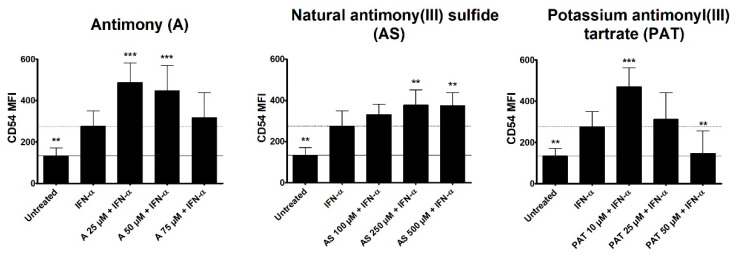
Effect of ACs on CD54 expression on THP-1 cells. THP-1 cells were pre-treated with either A, AS or PAT for 2 h before stimulation with 10 ng/mL IFN-α for 24 h. Afterwards, expression was analyzed by flow cytometry after staining CD54 with a specific antibody coupled to the fluorophore APC. Results are shown as mean fluorescence intensity (MFI) ± SD of 4 independent experiments. Statistical analysis was performed using one-way ANOVA with Newman–Keuls post-test comparing the samples to Pso stimulation. ** *p* ≤ 0.01, *** *p* ≤ 0.001.

Type I interferon (IFN) signaling, especially IFN-α, has been shown to be activated in lesional skin of psoriasis patients. However, keratinocytes as the main affected cells in psoriasis are not responding to IFN-α stimulation to create a psoriasis-like phenotype in vitro [36]. Nevertheless, the importance of IFN-α in psoriasis pathogenesis is unquestionable as cancer and hepatitis C patients receiving IFN-α as therapy have been seen to develop or exacerbate psoriasis upon treatment initiation [37,38]. Plasmacytoid DCs have been shown to be a main producer of IFN-α in psoriasis, leading to the activation and maturation of myeloid DCs that produce cytokines leading to activation of T cells [39]. We therefore analyzed a potential effect of ACs on DC activation. The human monocytic cell line THP-1 is often employed as a surrogate cell line for DCs and was stimulated with IFN-α with or without a pre-treatment with ACs and the surface expression of the activation marker CD54 was analyzed using flow cytometry. CD54, also known as ICAM-1, is an intercellular adhesion molecule expressed by a variety of cells. It is especially important in antigen-presenting cells (APCs) such as DCs, where it is crucial for the formation of the immunological synapse by binding to lymphocyte-function-associated antigen-1 (LFA-1) expressed by T cells. While DCs in steady state already express certain levels of CD54, surface expression greatly increases upon activation making it a suitable surrogate marker for the activation of DCs (Sheikh und Jones 2008). Both A and AS led to an increased mean fluorescence intensity (MFI) of CD54 in comparison to IFN-α stimulation (Figure 5). However, the highest tested concentration of A showed a tendency towards a reduced MFI. While 10 µM of PAT also led to a higher CD54 expression, increasing concentrations resulted in a reduced MFI of CD54 with 50 µM inhibiting CD54 to the level of untreated cells.

**Figure 6:**
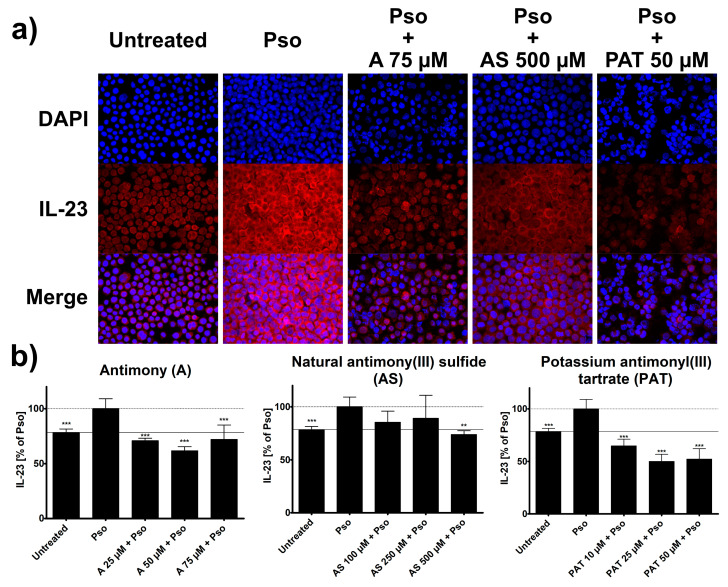
IFN-α-induced IL-23 expression in THP-1 cells is reduced by treatment with ACs. THP-1 cells were pretreated with A, AS or PAT for 2 h before stimulation with 10 ng/mL IFN-α for 24 h. Afterwards, cells were transferred to a glass slide using a cytocentrifuge. Cells were fixed, permeabilized and IL-23 was stained using a specific antibody. After visualization of IL-23 with secondary antibody coupled to Alexa 555 (red) and DAPI staining of the nucleus. (**a**) Representative pictures of IL-23 staining in untreated, Pso-treated and AC treated cells. (**b**) IL-23 expression was analyzed using the Intensity Ratio Nuclei Cytoplasm Tool (RRID:SCR_018573) in ImageJ. Data shown as mean ± SD of four independent experiments with two pictures per experiment. Statistical analysis was performed using column statistics with one-sample *t*-test comparing the samples to Pso stimulation. ** *p* ≤ 0.01, *** *p* ≤ 0.001.

Once active, DCs in psoriasis start to secrete a variety of pro-inflammatory mediators that lead to the activation of cells from both the innate and adaptive immune system. At this point, IL-23 is one of the key cytokines involved in psoriasis pathogenesis, as it is responsible for the differentiation of IL-17 producing T cells. Its expression by DCs is increased in lesional skin of psoriasis patients [5] and the success of therapies targeting IL-23, such as the monoclonal antibody ustekinumab, confirms its importance for the pathogenesis of the disease. Therefore, we analyzed potential effects of ACs on the IFN-α-induced expression of IL-23 in DCs using ICC. Just like in psoriasis, treatment of THP-1 cells with IFN-α led to an increased staining of IL-23 in these cells (Figure 6a).

The staining was decreased when cells were pre-treated with different concentrations of A, AS or PAT and analysis of staining intensity showed a clear reduction of IFN-α-induced IL-23 expression by these ACs (Figure 6b). Once again, PAT was already effective at relatively low concentrations starting at 10 µM and could reduce IL-23 expression way below the level of untreated cells. Pre-treatment with A resulted in similar reductions of IL-23 expression while AS was only effective at the highest of the tested concentrations. Lactose treatment had no effect on IL-23 expression (data not shown).

Taken together, the tested substances confirm their anti-psoriatic potential in IFN-α-treated THP-1 cells. Again, PAT shows the most prominent effects in both DC activation and IL-23 expression.

## 3. Discussion

Over the last years, a variety of new drugs has been developed to treat psoriasis. While monoclonal antibodies (biologicals) are very effective, they are only used in patients suffering from moderate to severe forms of psoriasis with high PASI scores or comorbidities such as psoriatic arthritis. These biologicals have systemic effects on the immune system, may have adverse effects (i.e., the TNF-inhibitors) and are quite expensive. Patients with mild to moderate forms of psoriasis make up most of the cases and do not qualify for these drugs. They require other types of treatment, typically applied directly to the skin. Commonly used substances include vitamin D3, corticosteroids or dithranol [40]. While they can temporarily lead to an improvement of the symptoms, adverse effects can limit their use. Long-term treatment with topical corticosteroids can cause skin fragility [41], while dithranol, especially in combination with other substances such as salicylic acid, can cause chemical burns [42] and should ideally be applied by trained nursing staff or physicians in an inpatient setting. New therapeutical options that could replace or reduce the use of these drugs are therefore needed. ACs could be such an alternative. Although a carcinogenic potential of ACs has been discussed, cancerogenic effects due to oral uptake of ACs have not been confirmed. A study described an association between the occurrence of lung cancer and chronic antimony inhalation in smelter workers [43]. However, the authors were critical of their results due to a list of confounders in this type of workplaces. A study analyzing the genotoxic properties of ACs found that the only evidence hinting towards any effect in that direction is the induction of oxidative stress that may lead to DNA damage. However, an in vitro reporter system did not sense DNA damage induced by ACs but found oxidative stress [44]. Interestingly, oxidative stress through the induction of ROS production is also one of the main modes of action of dithranol [45]. Dithranol is the most effective topical treatment of psoriasis to date, leading to the death of proliferating keratinocytes as a result of disturbed energy delivery due to interference with mitochondrial function [46].

To analyze potential anti-psoriatic effects of ACs, keratinocytes derived from lesional skin of patients would be the ideal model. However, isolation and cultivation of keratinocytes derived from biopsies of psoriatic lesions was only successful in a fraction of all our previous attempts. Similarly, other studies had the same problem, receiving cell cultures from only 10% of all psoriasis biopsies [47]. Furthermore, psoriasis keratinocytes isolated from lesional skin re-differentiated and lost their psoriatic phenotype during cell culture [48]. Therefore, our in vitro psoriasis model using primary keratinocytes isolated from the skin of healthy donors treated with a combination of IL-17A, IL-22 and TNF-α provides an interesting substitute model that has already proven its capability [23].

We used concentrations of ACs with a maximal cytotoxicity of 25%, a value that limits the probability that potential anti-psoriatic effects are merely mediated by cell death. In addition, effective AC concentrations that have been determined in vitro allow the calculation of effective concentrations for in vivo applications. The concentrations of ACs determined using the LDH release assay have shown promising results in modulating the expression of different genes important for the pathogenesis of psoriasis. *DEFB4A* was the most responsive gene to ACs. Both A and PAT reduced its expression by 90% while AS reduced it by 75% These effects were in the same range as with dithranol, which we have used as positive control [23]. While its role as an AMP is widely known, BD2 also plays an important role in the activation of pro-inflammatory pathways and immune cells. In psoriasis, it not only enables the complex formation of AMPs with self-nucleotides resulting in the activation of DCs via TLR recognition, but it also acts directly on keratinocytes by inducing pro-inflammatory cytokine secretion, proliferation and migration [49]. Its downregulation by ACs is in line with the finding that BD2 is also reduced in patients after anti-IL-17A therapy using secukinumab [25]. *KRT1* is also a gene that was massively downregulated in psoriasis-like PHKs. Interestingly, this not only indicates reduced differentiation in keratinocytes; KRT1 has also been shown to be a crucial player in the maintenance of skin integrity, and its loss or mutation leads to increased levels of IL-18 as well as alarmins such as S100A8 [50]. A therapy increasing its expression would therefore improve psoriasis. In our setting, PAT, but not A and AS, was able to increase the expression of *KRT1*. The expression of keratin 17 (*KRT17*) was also modulated by ACs. *KRT17* was downregulated by A and PAT to the level of untreated cells or even below. This was also observed with corticosteroids or dithranol [51,52] indicating a reduction in psoriasis-associated hyperproliferation. Similarly, in a SCID-hu xenogeneic transplantation model, siRNA-mediated inhibition of KRT17 was shown to reduce typical histological features of psoriasis such as increased epidermal thickness [53]. This confirms the importance of KRT17 as a target for psoriasis therapy. It is not only important for proliferating keratinocytes but also a target for autoreactive T cells [54,55], partly due to peptide sequence similarities with streptococcal M proteins [56]. The KRT17-mediated hyperproliferation is a very energy-consuming process for keratinocytes. Associated with increased metabolism, *GLUT1* expression is upregulated in these cells [30]. Inhibiting *GLUT1* using ACs might therefore be an option to limit the energy transfer to keratinocytes and reduce proliferation. However, the ability of ACs to reduce *GLUT1* expression was rather limited. Only the lowest concentration of PAT was able to cause a significant *GLUT1* downregulation while all other doses led to an increased expression. At first glance, this seems to be the opposite of the desired effect of AC treatment. However, the upregulation of *GLUT1* expression might indirectly be caused by the effect of ACs on glucose and its catabolism. It has been shown earlier that the effect of ACs against *Leishmania tropica* is mediated by an inhibition of glucose uptake into the cells [57], while ACs reduced glycolysis in *Schistosoma mansoni* [58]. Given that ACs may have similar effects on mammalian cells, the massive upregulation of *GLUT1* expression might be an effort of the keratinocytes to compensate the lack of glucose metabolism. In conclusion, from our experiments, 10 µM of PAT could be a concentration where the anti-psoriatic effect is already present while inhibition of glucose uptake or metabolism is not present.

Besides the phenotypical changes of hyperproliferation and reduced differentiation, keratinocytes themselves are part of the pro-inflammatory processes in the skin of psoriasis patients. Two important signaling pathways in psoriatic inflammation are NF-κB and STAT3 [32,33]. Both pathways are activated in our psoriasis-like PHKs after Pso-treatment. While AS has no effect on this activation, A shows a tendency towards an inhibition of the nuclear translocation of the p65 subunit of NF-κB as well as phosphorylation and nuclear translocation of STAT3 at higher concentrations. However, PAT showed a great efficiency in reducing the activation of both pathways, even at 25 µM. These results are again comparable to effects we have seen in cells treated with dithranol. Inhibition of these pathways offers an interesting opportunity to reduce disease severity in psoriasis [59,60]. Besides the inhibition of inflammation, the inhibition of STAT3 can also reduce proliferation of keratinocytes as *KRT17* expression is upregulated by IL-17A in an STAT3-dependent mechanism [61]. It has been shown that the inhibition of NF-κB and STAT3 in murine psoriasis models using benzo[b]thiophen-2-yl-3-bromo-5-hydroxy-5H-furan-2-one (BTH) [62] and inhibition of STAT3 in psoriasis patients using ochromycinone [63] is able to reduce psoriatic skin inflammation. 

One of the downstream events of the activation of NF-κB and STAT3 is the production and secretion of pro-inflammatory cytokines. Important examples of such cytokines produced by keratinocytes in the skin of psoriasis patients are IL-6 and IL-8 [34,35]. Both cytokines have been targets in the development of new therapeutical options. There have been studies analyzing potential effects of anti-IL-6/8 therapy. The success of the monoclonal antibody tocilizumab targeting IL-6 in palmoplantar pustular psoriasis and psoriatic arthritis [64,65] has been overshadowed by the exacerbation of psoriasis in some of the treated patients [66]. Although both cytokines are important in a broad range of immune functions, their systemic inhibition often results in infections. Therefore, the local reduction of cytokine secretion by topical application of PAT might be advantageous, as PAT was the only AC that reduced the secretion of both IL-6 and IL-8 in Pso-stimulated keratinocytes. This effect of PAT is most probably mediated by the inhibition of NF-κB and STAT3 signaling. It was observed within the same concentrations range as with the positive control dithranol in a previous study. Surprisingly, although A did not reduce the nuclear translocation of p65 or pSTAT3, it was able to reduce IL-6 secretion. This might indicate an alternative way of activating IL-6 expression.

DCs initiate the pro-inflammatory signaling cascade in psoriasis, ultimately resulting in pro-inflammatory activation of keratinocytes. Upon their activation by AMP/self-nucleotide-complexes, plasmacytoid DCs start to secrete IFN-α, leading to the activation of monocytic DCs. These cells then secrete a variety of cytokines, leading to the activation of different cells of the immune system. Here, only PAT was able to reduce the activation status of DCs, while A and AS led to an increased CD54 surface expression. As these are both ACs that are not soluble in the cell culture medium, they might cause an increased activation of DCs in a similar manner that has been observed for urban particulate matter [67]. However, an initial upregulation has also been seen with the soluble PAT. As the concentrations were initially determined in psoriasis-like PHKs, higher levels of ACs might be necessary to achieve effects when using A or AS.

In contrast to the activation, all ACs influenced pro-inflammatory cytokine production. One of the most important cytokines in psoriatic inflammation is IL-23. It is regarded as the psoriatic master cytokine that promotes the differentiation, proliferation and maintenance of T_H_17 cells and is highly expressed in psoriatic skin [5]. The importance of IL-23 in psoriatic inflammation is confirmed by the very high efficacy of therapies targeting this cytokine. Monoclonal antibodies such as the p40 inhibitor of IL-12 and IL-23, ustekinumab, and the p19 inhibitors of IL-23, guselkumab, tildrakizumab and risankizumab, are very effective in the treatment of severe forms of psoriasis [9,68,69]. While all three tested substances were effective in inhibiting IL-23 expression, PAT was the most effective, reducing IL-23 expression even below the level of untreated cells. We conclude that the inhibitory effect of all three ACs on IL-23 expression in IFN-α-treated THP-1 cells supports the potential of ACs as topical treatment for psoriasis. As a reduction of this key cytokine might be enough to achieve an effect in the treatment of psoriasis, a reduction of the activation status of DCs might not be necessary.

While our study provides promising results in favor of the use of ACs in the treatment of psoriasis, this is only the first step towards the use of these substances in patients. As shown by Mathes et al. [70], monocultures of human cells are useful tools for the initial steps in drug development for dermatological disorders. While these models can provide evidence of the mode of action of the investigated substances, they lack the complexity of skin tissue consisting of a variety of cell types. In the human system, the next step would be the use of organotypic skin models or explants from human skin. In addition, testing the ACs in vivo using murine models of psoriasis, induced, for example, by imiquimod or IL-23, could give insight into the effects of ACs in a more physiological setting. The advantage of ACs is that two of them are already used in commercially available ointments containing 0.4% of either A or AS. These products are used in anthroposophic medicine for the treatment of dermatological disorders such as eczema. The 0.4% concentration is well tolerated by patients and could also be used for an initial assessment of the effect of ACs in psoriasis patients.

## 4. Materials and Methods

### 4.1. Antibodies and Reagents

The following antibodies were used for immunocytochemical staining: anti- p65 antibody (F-6, 1:50, Santa Cruz, Heidelberg, Germany), anti-phosphoSTAT3 antibody (Tyr705; 1:200, Cell Signaling Technologies, Leiden, The Netherlands) and anti-IL-23 antibody (1:100, Abcam, Cambridge, UK). The secondary antibodies Alexa Fluor 555 goat anti-mouse IgG and Alexa Fluor 555 donkey anti-rabbit IgG were from Thermo Fisher Scientific (1:500, Dreieich, Germany). For flow cytometry, an anti-CD54 antibody with conjugated APC was used (1:50, BD Bioscience, Heidelberg, Germany). IL-22, IL-17A and TNF-α were purchased from Peprotech (Rocky Hill CT, USA). DAPI (40,6-Diamidino-2-phenylindole dihydrochloride) and IFN-α were purchased from Sigma-Aldrich GmbH (Taufkirchen, Germany). Antimony (Stibium metallicum, D1 trituration in lactose), natural antimony(III) sulfide (D1 trituration in lactose) and potassium antimonyl(III) tartrate were provided by Weleda AG (Schwäbisch Gmünd, Germany) and were further diluted in cell culture medium.

### 4.2. Cell Culture

Primary human keratinocytes (PHKs) were isolated from healthy skin removed during reduction surgeries (approved by the ethics committee of the University Medical Center Freiburg, Certificate No EK432/18) and cultured according to the method of Rheinwald and Green [71]. They were kept in Keratinocyte-SFM medium (Thermo Fisher, Darmstadt, Germany) at 37 °C in a humidified atmosphere with 5% CO_2_ and were passaged regularly before reaching confluence to keep them in a basal/early differentiation state. The cells were used up to passage 5. To generate psoriasis-like PHKs, cells were incubated with psoriasis cytokines (IL-17A, IL-22 and TNF-α, 20 ng/mL each) for 24 h. THP-1 cells (donor: Cell line services, Eppenheim, Germany, source: BIOSS, University of Freiburg, Freiburg, Germany) were kept in RPMI1640 supplemented with 4 mM L-glutamine, 50 µM β-mercaptoethanol, 25 mM HEPES and 10% FCS.

### 4.3. Cell Viability Assay

To determine the cytotoxic effects of the tested ACs, the release of lactate dehydrogenase from the treated cells was measured using the Cytotoxicity Detection Kit (Roche, Grenzach-Wyhlen, Germany). To calculate the level of toxicity, an untreated control was set to 0% toxicity and a cell death control treated with 1% Triton X-100 was set as 100% toxicity.

### 4.4. RNA Extraction, cDNA Synthesis and qPCR

The expression of DEFB4, KRT1, KRT17 and GLUT1 was analyzed using real-time qPCR. Total RNA was isolated from PHKs using TRIzolTM (Thermo Fisher, Darmstadt, Germany). Following isolation, 1 µg of total RNA was reverse transcribed to receive cDNA using the iScript cDNA Synthesis Kit (BIO-RAD, Feldkirchen, Germany). Maxima SYBR Green qPCR Master Mix (Thermo Fisher, Darmstadt, Germany) was used to perform the qPCR on a Bio-RAD CFX96TM Real-Time System.

The following primers were used: huDEFB4: 5’-ACCACCAAAAACACCTGGAAG-3’ forward and 5’-ACCAGGGACCAGGACCTTTA-3’ reverse; huKRT1: 5’-GGCAGACATGGGGATAGTGTG-3’ forward and 5’-CTTGAGGGCATTCTCGCCA-3’ reverse; huKRT17: 5’-GAGATTGCCACCTACCGCC-3’ forward and 5’-ACCTCTTCCACAATGGTACGC-3’ reverse; huGLUT1: 5’-TCTGGCATCAACGCTGTCTT-3’ forward and 5’-AAGGCAAGTGTCTCGACAGG-3’ reverse; huACTB: 5’-GACCCCGTCACCGGAGTCCA-3’ forward and 5’-CGAGCACAGAGCCTCGCCTTT-3’ reverse.

The relative gene expression was determined using the comparative CT method (2^−ΔΔCt^) [27] with ACTB as the reference gene. As PHKs were isolated from skin biopsies of different donors, the response to the stimulation varied in its strength due to donor variation. For example, induction of DEFB4A expression by Pso-stimulation ranged from 1209- to 2405-fold and KRT17 expression ranged from 1.4- to 2.9-fold. However, the effects of the ACs always showed similar trends in comparison to those values. To enable better comparison of the results, the expression of Pso-treated cells was set as 100% expression and the results of the other sample were calculated in comparison.

### 4.5. Immunocytochemistry (ICC)

PHKs were seeded on 12 mm collagen-coated coverslips in a multiwell plate and pre-treated with ACs for 2 h before the stimulation with 20 ng/mL of IL-17A, IL-22 and TNF-α for 30 min. THP-1 cells were pre-treated with ACs for 2 h before the stimulation with 10 ng/mL of IFN-α for 24 h, then removed from the multiwell plate and transferred to a glass slide using a cytocentrifuge. Afterwards, the cells (PHKs or THP-1s) were fixed using 4% formaldehyde at RT for 10 min and permeabilized with methanol at −20 °C for 10 min. Subsequently, the cells were blocked with 5% BSA and 0.1% Tween-20 in PBS for 1 h at RT and incubated with the primary antibodies overnight at 4 °C. Then, secondary antibodies were added for 1 h at RT and DAPI was used for nuclear control staining. The mean intensity of the staining was measured using the Intensity Ratio Nuclei Cytoplasm Tool (RRID:SCR_018573) in ImageJ.

### 4.6. ELISA

Concentrations of IL-6 and IL-8 in the cell culture supernatants were measured using high sensitivity ELISA kits (BD, San Jose, California CA, USA). The assay was performed according to the manufacturer’s protocol. As with the qPCRs, variability of the results between cells from different donors was high. Therefore, results were also normalized to 100% for Pso-treated cells. IL-6 levels secreted from untreated cells ranged from 2 pg/mL to 10 pg/mL, Pso-treated cells ranged from 40 pg/mL to 63 pg/mL. IL-8 secretion from untreated cells was between 120 pg/mL and 336 pg/mL and from Pso-treated cells between 2752 pg/mL and 8977 pg/mL.

### 4.7. Flow Cytometry

THP-1 cells were seeded in 96-well plates and pre-treated with the indicated concentrations of A, AS or PAT for 2 h. Afterwards, cells were stimulated by adding 10 ng/mL IFN-α for 24 h. Finishing pre-treatment and stimulation, cells were washed, stained with an APC-coupled antibody specific for CD54 (BD Bioscience, Heidelberg, Germany). Immediately before recording, DAPI was added to exclude dead cells from the subsequent analysis. The raw data were collected using a FACSCanto II (BD Bioscience, Heidelberg, Germany) and then analyzed using FlowJo 10 (FlowJo LLC, Ashland, Catlettsburg, KY, USA).

### 4.8. Statistical Analysis

Data analysis was performed using GraphPad Prism version 6.0 software (GraphPad Software, San Diego, CA, USA). Significant statistical differences were evaluated using one-way analysis of variance (one-way ANOVA) followed by the Newman–Keuls Test or the column statistic with one sample *t*-test (when Pso-stimulated cells were set as 100%). *p*-values of <0.05 were considered statistically significant. * *p* ≤ 0.05, ** *p* ≤ 0.01, *** *p* ≤ 0.001.

## 5. Conclusions

In summary, ACs showed prominent anti-psoriatic effects in our in vitro psoriasis model at subtoxic concentrations. ACs reduced the expression of psoriasis genes in keratinocytes as well as activation and IL-23 expression in THP-1 cells. Our study confirms the potential of ACs in the topical treatment of psoriasis.

## Data Availability

The data presented in this study are available on request from the corresponding author.

## Sample Availability

Samples of the compounds are available from the authors.
